# Factors Affecting Residual Stone Rate, Operative Duration, and Complications in Patients Undergoing Minimally Invasive Percutaneous Nephrolithotomy

**DOI:** 10.3390/medicina58030422

**Published:** 2022-03-13

**Authors:** Mladen Doykov, Gancho Kostov, Katya Doykova

**Affiliations:** 1 Department of Urology and General Medicine, Medical Faculty, Medical University of Plovdiv, 4001 Plovdiv, Bulgaria; 2 Department of Urology, University Hospital “Kaspela”, 4001 Plovdiv, Bulgaria; 3 Department of Special Surgery, Medical Faculty, Medical University of Plovdiv, 4001 Plovdiv, Bulgaria; gancho.kostov@mu-plovdiv.bg; 4 Department of Surgery, University Hospital “Kaspela”, 4001 Plovdiv, Bulgaria; 5 Department of Diagnostic Imaging, Medical Faculty, Medical University of Plovdiv, 4001 Plovdiv, Bulgaria; katya.doykova@mu-plovdiv.bg; 6 Department of Diagnostic Imaging, University Hospital “Kaspela”, 4001 Plovdiv, Bulgaria

**Keywords:** kidney stones, minimally invasive percutaneous nephrolithotomy, residual stone rate, operative duration, hospital stay, complications

## Abstract

*Background and objectives*: Although minimally invasive percutaneous nephrolithotomy (MPCNL) has demonstrated its efficacy, complete stone clearance was not always achieved, necessitating a second procedure. The purpose of this study was to evaluate factors associated with residual stone rate, operative duration, complications, and hospital stay, in order to develop algorithms for pre-operative prognosis and planning. *Materials and Methods:* This retrospective study involved 163 Bulgarian patients who underwent MPCNL with Holmium: YAG lithotripsy for the treatment of kidney stones. Patients were considered stone-free if no visible fragments (<3 mm) were found on nephroscopy at the end of the procedure, as well as on postoperative X-ray and abdominal ultrasound on the first postoperative day. *Results:* Immediate postoperative stone-free outcome was attained for 83.43% of the patients (136/163). Residuals were associated with staghorn stones (OR = 72.48, 95% CI: 5.76 to 91.81); stones in two locations (OR = 21.91, 95% CI: 4.15 to 137.56); larger stone size (OR = 1.12, 95% CI: 1.006 to 1.25); and higher density (OR = 1.03, 95% CI:1.005 to 1.06). The overall categorization accuracy for these factors was 93.80%, AUC = 0.971 (95% CI: 0.932 to 0.991), 89.71% sensitivity, and 96.30% specificity. Predictors of prolonged operative duration were staghorn stones and volume, R-square (adj.) = 39.00%, *p* < 0.001. Longer hospitalization was predicted for patients with hydronephrosis and staghorn stones, R-square (adj.) = 6.82%, *p* = 0.003. Post-operative complications were rare, predominantly of Clavien-Dindo Grade 1, and were more frequent in patients with hydronephrosis. We did not find a link between their occurrence and the outcome of MPCNL. *Conclusions:* Staghorn stones and stones in more than one location showed the strongest association with residual stone rate. Staghorn stones and larger volume were linked with a longer operative duration. Hydronephrosis increased the risk of complications and longer hospitalization.

## 1. Introduction

In primary care, kidney stone disease is a common occurrence. The pharmaceutical therapy of urolithiasis was determined by the chemical composition of the stone, which rarely results in the desired release of the calculi. Minimally invasive percutaneous nephrolithotomy (MPCNL) is one of several advanced techniques for disintegration and removal of renal calculi. Since it was first introduced by Jackman et al. [1], the procedure has been modified with the purpose of increasing its efficacy and safety. The standard percutaneous nephrolithotomy (PCNL) uses a nephrostomy tract of 24 Fr to 34 Fr, whereas the MPCNL (aka mini-PCNL) is performed with a mini endoscope of 12 Fr via a smaller percutaneous tract of 11 Fr to 20 Fr [2]. The minimized access is associated with less blood loss, fewer blood transfusions, hematuria, reduced incidence of organ damage, and shorter hospital stay [3,4,5,6,7].

A meta-analysis on comparative research between PCNL and MPCNL established a higher stone-free rate (SFR) after MPCNL [8]. Studies on MPCNL have provided confirmatory evidence about its efficacy and safety in the treatment of simple and complex renal stones [9,10].

Various factors associated with operative duration, immediate postoperative stone-free rate (SFR), surgical complications, and hospital stay have been examined in univariate and multivariate analyses. Stone burden, density, type, location, and hydronephrosis are commonly reported factors, with an impact on SFR and operative duration [11,12,13]. Complications have been linked to higher ASA scores, staghorn stones, positive urine cultures, multiple tracts, and others [13].

Although minimally invasive percutaneous nephrolithotomy (MPCNL) has demonstrated its efficacy, complete stone clearance is not always achieved with a single MPCNL, necessitating further procedures [9,10,11,12,13]. The purpose of this study was to identify risk factors linked to residual stone, prolonged operative duration, complications, and lengthened hospital stay, in order to develop algorithms for pre-operative prognosis and planning. Reviewing the literature, we also observed that the majority of the studies on the factors influencing the efficacy of MPCNL were conducted with patient populations in Asia, primarily China [8]. The addition of data from a new population of Bulgarian patients was determined to broaden the scope of the previous investigations.

## 2. Materials and Methods

The present study used data from 163 patients with renal stones who underwent MPCNL in the department of Urology at the University Hospital “Kaspela” in Plovdiv, Bulgaria, between January 2017 to January 2020. The research protocol was reviewed and approved by the Institutional Review Board (IRB No:21- 2015-12-16). Before the treatment, all patients signed a written consent form upon admission to the hospital, in compliance with the ethical principles specified in the World Medical Association Declaration of Helsinki, revised in 2000, Edinburgh.

Inclusion criteria were solitary stones with high density, multiple renal stones, partial staghorn stone, complete staghorn stone, inability to conduct ESWL, inability to conduct fURS. Excluded, were patients with severe cardiac or pulmonary dysfunction, coagulation disorders, uncontrolled hypertension or diabetes mellitus, acute and chronic renal failure, acute pyelonephritis, congenital anomalies of the kidneys, and renal tumors. Based on the urine culture results, all patients with urinary tract infections received preoperative antibiotics for at least 5 days. Patients without signs of infection received perioperative antibiotic prophylaxis. The antibiotic administration continued until the removal of the percutaneous nephrostomy tube. Preoperatively, a CT scan was performed on every patient, in order to obtain accurate data about the location and characteristics of the stones. The standard Digital Imaging and Communications in Medicine (DICOM) viewing software (RadiAnt Dicom Viewer Version 3.4.2, 2016) [14] was used to establish precise stone measurements. Stone volume was calculated using an ellipsoid formula, as recommended in the guidelines of the European Association of Urology: stone volume = π∗l∗w∗d∗0.167, where l = length, w = width, and d = depth [15].

Each of the 163 surgeries was carried out under epidural anesthesia. A 12 Fr. small nephroscopic system (Karl Storz SE & Co. KG, Tuttlingen, Germany) and a modified 16 Fr. access aspiration shaft (Clear Petra, Well Lead Medical Co., Ltd, Guangzhou, China) were used. After the retrograde insertion of a 6 Fr. ureteral catheter to contrast the renal collector system and its fixation to the Foley catheter, the patients were placed in a supine position atop an inflatable cushion which helped secure a 15–20 degree slant, reducing the risk of perforation of the colon.

Percutaneous access was achieved with an 18G needle under combined (ultrasound and X-ray) control. Irrespective of the size and location of the stone, a single tract MPCNL was performed in all cases; however, in some of the patients with residual stones, we made an additional tract to perform a second MPCNL. A single dilatation with a 10 Fr. nitinol dilator was applied via the indwelling hydrophilic guidewire (0.035 inch) and then a 16 Fr. access aspiration shaft was introduced. The stones were defragmented using a Ho:YAG laser (H-30, COOK MEDICAL LLC Bloomington, IN, USA), with a laser fiber size of 550 µm.

The procedure was carried out under constant visibility, and the stone fragments were evacuated by aspiration through the modified access shaft. The retrograde contrast catheter was withdrawn from the kidney at the conclusion of the procedure. The access shaft was slowly removed from the patient. At the end of every procedure, a 14-Fr nephrostomy tube was inserted to ensure kidney drainage, as well as an inlet for a second surgery in those cases where residual fragments were detected. In the latter cases, extracorporeal shock wave lithotripsy (ESWL) and flexible ureterorenoscopy were used for smaller residuals, and a second MPCNL was performed for bigger residuals. If no visible fragments (<3 mm) were found on nephroscopy at the end of the surgery and on the postoperative abdominal ultrasound and X-ray, the patient was considered stone-free.

A CT scan was performed on the patients with non-opaque stones and when the results of the other methods of examination were inconclusive. The duration of the surgery was recorded from the time of puncture of the kidney until the final placement of the nephrostomy tube. Surgical complications were recorded according to the Clavien-Dindo classification [16].

### Statistical Analysis

The data were analyzed using the statistical software IBM SPSS version 27 (2020) [17] and MedCalc version 21.014 (2021) [18]. Continuously measured and normally distributed variables were described through their mean values and standard deviations, and between-group comparisons were performed using the t-test for independent samples or one-way analysis of variance (ANOVA). Variables that were not normally distributed were described with the medians and interquartile ranges (IQRs). Between-group comparisons were carried out through the Mann–Whitney U test (two groups) or the Kruskal–Wallis test (more than two groups). P-values were adjusted by the Bonferroni correction for multiple tests. Binary and categorical data were presented in frequencies and percentages, and associations were established through the Chi-square test and Fisher’s exact test. Binary logistic regression was used to identify factors with significant impacts on the duration and immediate postoperative outcome of MPCNL. A receiver operating characteristic (ROC) curve was used to assess the accuracy of prognostic factors and models. All statistical tests were two-tailed and performed at a level of significance *alpha* = 0.05.

## 3. Results

The patients were categorized into two subgroups according to the immediate SFR: 136 (83.40%) stone-free patients and 27 (16.60%) with residual stone (Table 1). The mean age was 53.60 ± 13.47 years, 54.14 ± 13.59 years in the stone-free group, and 50.85 ± 12.79 years in the group with residual stone, with no significant age difference (*p* = 0.246). Male patients constituted 63% of the entire sample, 62.50% of the stone-free group, and 66.70% of the patients with residual stone (*p* = 0.828). Based on the patients’ BMI, they were categorized into the following categories: normal weight from 18.5 kg/m^2^ to 24.9 kg/m^2^; overweight from 25 kg/m^2^ to 29.9 kg/m^2^; obese ≥ 30 kg/m^2^. Underweight patients (>18.5 kg/m^2^) were not present in any of the two groups, and the distribution among the other three categories did not differ significantly, *p* = 0.778.

The groups did not differ significantly in operative risk (*p* = 0.972), hematuria history (*p* = 0.801), the presence of hydronephrosis (*p* = 0.675), HGB (*p* = 0.995), WBC (*p* = 0.583), PLT (*p* = 0.747), INR (*p* = 0.914), creatinine (*p* = 0.515), urea (*p* = 0.875), and glucose (*p* = 0.733). The patients with residual stone had a longer hospital stay (median 5 days) than those in the SFR group (median 4 days), but the difference was not significant (*p* = 0.123). The mean operative time was 61.91 ± 26.29 min, with a significantly longer duration in the group with residual stone (*p* < 0.001).

**Figure 1:**
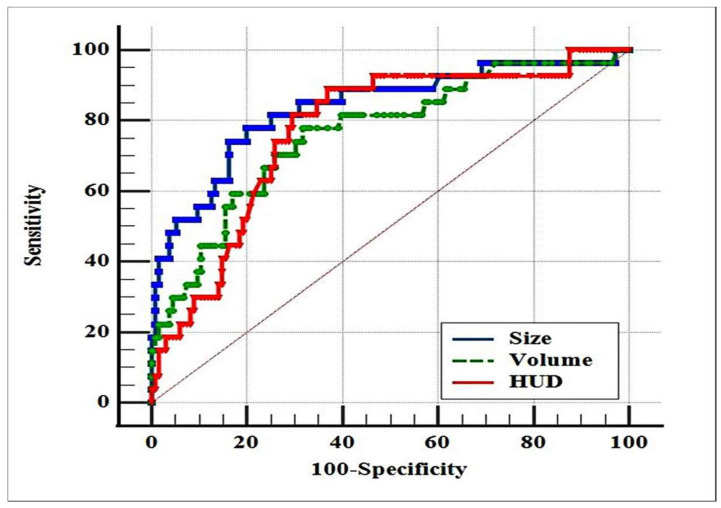
Receiver operating characteristic curves for stone size, volume, and density versus stone-free rate after MPCNL.

The ROC curve analysis showed AUC = 0.836 (95% CI: 0.770 to 0.889, *p* < 0.001) for size; AUC = 0.764 (95% CI: 0.691 to 0.827, *p* < 0.001) for volume; and AUC = 0.772 (95% CI: 0.699 to 0.834, *p* < 0.001) for density (Figure 1).

### 3.1. Factors Associated with Residual Stone

The majority of the patients (69.30%) were diagnosed with solitary stones, 20.90% with multiple stones, and 9.80% with staghorn stones. In the stone-free group, 83% had solitary stones versus 0.00% in the group with residual stone (*p* < 0.001). The patients with residual stone had either multiple or staghorn stones. Fifteen of the 16 staghorn stones were in the group with residual stones. The majority of the stone-free group (83.10%) had stones in one location (the pelvis or the calices), whereas 85.20% of the group with residual stones had stones in both locations (*p* <0.001). The patients with residual stone were associated with bigger stone size (*p* < 0.001), larger volume (*p* < 0.001), and higher density (*p* < 0.001) (Table 2).

In a multivariate binary logistic regression, stone size, stone density, stone burden, and stone location were shown as the best predictors of residual stone, χ^2^ = 94.202 (df. 4), *p* < 0.001, Nagelkerkes R2 = 74.08%. Larger stone size, higher density, staghorn stone, and stones in two locations (pelvis and calices) were associated with a significantly higher probability of residuals after MPCNL (Table 3):

P(0) = exp(Y′)/(1+exp(Y′)), where Y′ = −8.17 + 0.10 × stone size + 0.002 × stone density + 4.28 (if staghorn) or + 0 (if single or multiple) + 3.17 (if pelvis and calices) or + 0 (if either pelvis or calices) (1)

**Figure 2:**
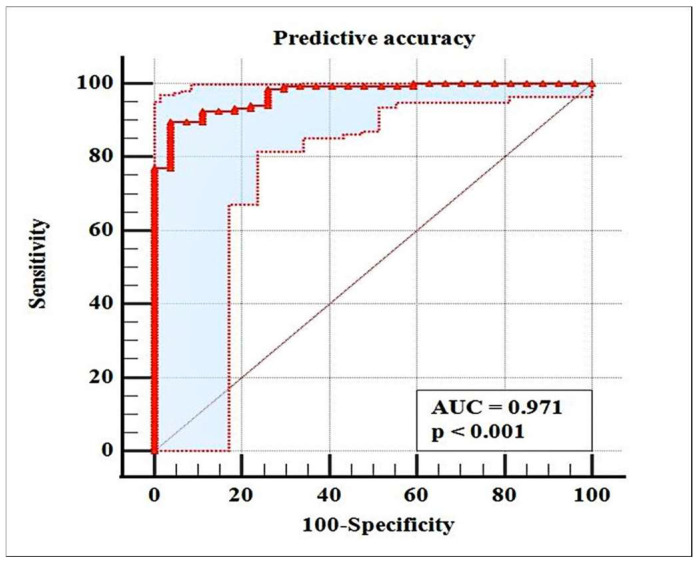
Accuracy of predicting residual stone with predictors stone size, density, burden, and location. The ROC curve is marked by the thick red curve. The lower and upper bounds of the 95% CI are indicated by thin red line curves on both sides of the ROC curve. The area of the 95% CI is colored in gray.

The predictive model was characterized by 93.80% correctly classified cases, AUC = 0.971 (95% CI: 0.932 to 0.991), sensitivity of 89.71%, and specificity of 96.30% (Figure 2).

### 3.2. Factors Influencing Operative Duration and Hospital Stay

The BMI categories were significantly associated with the operative duration (*p* = 0.037). The patients with normal BMI (18.5 kg/m^2^ to 24.9 kg/m^2^) had a shorter operative time, as compared to the overweight (25 kg/m^2^ to 29.9 kg/m^2^) and obese (>30 kg/m^2^) patients; however, the difference was significant only between the normal and overweight groups (*p* = 0.033). Hospital stay was not significantly associated with the BMI groups (*p* = 0.714).

Operative risk was not significantly associated with operative duration (*p* = 0.251) but showed a significant impact on hospital stay (*p* = 0.039). The patients with ASA 3 were characterized by a significantly longer median hospitalization duration in comparison with the ASA 1 category (*p* = 0.014). The other pairwise comparisons were not significant (ASA 1 versus ASA 2, *p* = 0.570; ASA 2 versus ASA 3, *p* = 0.105).

Stones in the pelvis were associated with a shorter operative duration compared to those in the calices or in both locations (*p* = 0.018). The difference was significant only between locations in the pelvis, and both pelvis and calices (*p* = 0.015). Stone location was not related to hospital stay (*p* = 0.984).

Stone size was another significant factor, affecting the duration of surgery (*p* < 0.001). The shortest median duration was associated with the small size stones (*p* = 0.004 vs. medium size; *p* < 0.001 vs. large size). Medium size stones took a shorter operative time as compared to large size calculi, but the difference was not significant (*p* = 0.141). The length of hospitalization was not associated with stone size (*p* = 0.624).

A significant association was found between stone burden and operative duration (*p* = 0.001). The surgery of staghorn stones took a significantly longer time as compared to solitary stones (*p* > 0.001) and multiple stones (*p* = 0.043). Stone burden was also significantly associated with hospital stay (*p* = 0.008). The patients with staghorn stones were hospitalized longer than the patients with solitary stones (*p* = 0.006) and with multiple stones (*p* = 0.028).

The patients with hydronephrosis were hospitalized longer than those without (*p* = 0.020). The presence of hydronephrosis was not associated with operative duration (*p* = 0.304). Stone density showed a significant, however feeble, positive association with operative duration (r^s^ = 0.252, *p* = 0.001) and no relation with hospital stay (*p* = 0.798). A moderate positive relation was found between stone volume and operative duration (rs = 0.471, *p* < 0.001). The length of hospitalization was not associated with stone volume (*p* = 0.209) (Table 4).

BMI, stone location, size, burden, density, and volume were entered as predictors in a multivariate linear regression analysis (Wald’s backward method). Stone volume (*p* < 0.001) and staghorn stone (*p* = 0.02) were shown as significant predictors, with R-square (adj.) = 39.00%, *p* < 0.001. The algorithm was expressed as follows:

Operative duration = 61.40 + 1.79 × Volume + 13.32 (if staghorn) or + 0 (if multiple or solitary)(2)

Operative risk, stone burden, and the presence of hydronephrosis were significantly associated with the length of hospitalization in the univariate analysis. Of them, stone burden and hydronephrosis were retained in the multivariate linear regression model as significant predictors of hospital stay, R-square (adj.) = 6.82%, *p* = 0.003.

Hospital stay = 4.60 + 1.499 (if staghorn) or + 0 (if solitary or multiple stones) +0.622 (if hydronephrosis YES) or + 0 (if hydronephrosis NO) (3)

### 3.3. Surgical Complications on the Clavien-Dindo Classification

**Figure 3:**
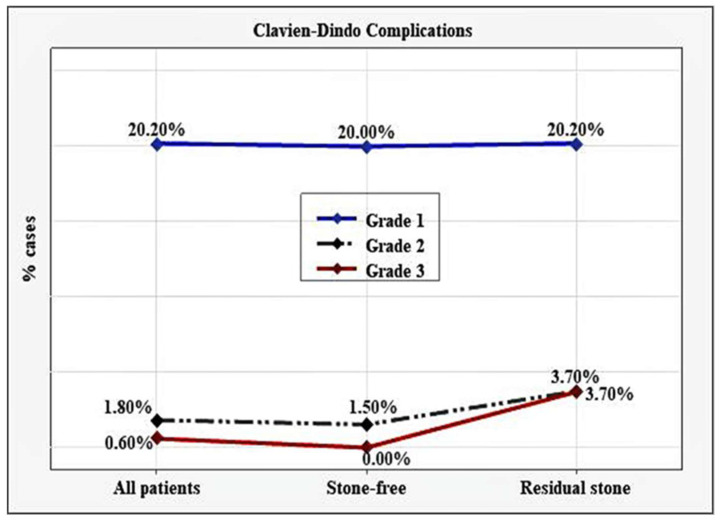
Postoperative complications on the Clavien-Dindo classification.

The overall complication rate was 22.69% (*n* = 37), of which 20.20% (*n* = 33) were Grade 1, 1.80% (*n* =3) Grade 2, and 0.60% (*n* = 1) Grade 3. The post-operative complications were not significantly associated with the outcome of MPCNL (*p* = 0.116). Grade 1 complications were observed in 20% (*n* = 27) of the stone-free group and in 22.20% (*n* = 6) of the patients with residual stones. All of them were cases with fever >38 °C. Grade 2 complications were found in two cases (1.5%) with gross hematuria in the SF group, and one case with blood transfusion (3.7%) in the group with residual stones. Grade 3 complications included one case (3.7%) with obstruction, requiring double-J stent placement, in the group with residual stones, and it did not occur in the SF group (Figure 3).

We found a significant association between the presence of hydronephrosis and postoperative complications: 29.20% (*n* = 27) in the group with hydronephrosis versus 13.40% (*n* = 9) in the group without hydronephrosis (*p* = 0.018). The three cases with Grade 2 and the one case with Grade 3 complications were in the group with hydronephrosis.

The other patient/stone variables were not associated with post-operative complications: age (*p* = 0.407); BMI (23.30% in patients with normal BMI; 25.90% in overweight; 17.20% in obese, *p* = 0.509); stone volume (*p* = 0.339); stone density (*p* = 0.922); stone size (*p* = 0.407); stone burden (37.50% in patients with staghorn; 32.40% in patients with multiple stones; 17.70% in patients with single stones, *p* = 0.067); and stone location (23.60% pelvis; 17.80% calices; 26.10% in both locations, *p* = 0.620).

## 4. Discussion

Our results support previous reports about the efficacy and safety of MPCNL for surgical treatment of renal stones. In the present study, the immediate postoperative SFR was 83.43%, which falls within the reported range of statistics [9,10,11,12,13]. Nevertheless, in 16.57% of the cases, a stone-free outcome was not achieved the first time, necessitating a follow-up surgery. The multivariate binary logistic analysis identified staghorn stone as the highest risk factor for residuals (OR = 72.48). Fifteen (93.75%) of the 16 patients with staghorn stones did not obtain full elimination in the first surgery. Stones in two locations (OR = 21.91), larger stone size (OR = 1.12), and higher density (OR = 1.003) were also associated with an increased chance for residuals. Together, the four predictors had a classification accuracy of 93.80%, sensitivity of 89.71%, and specificity of 96.30%. Stone volume was associated with residuals in the univariate analysis but did not contribute to the prognostic model.

**Figure 4:**
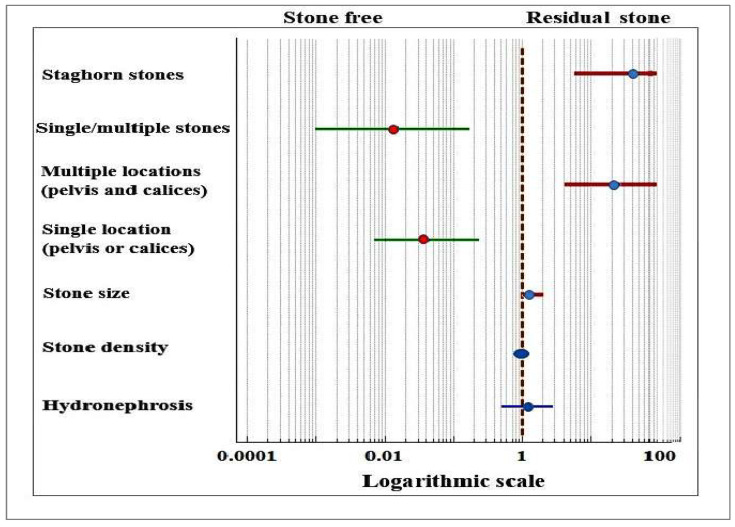
Forest plot of the odds ratios and 95% confidence intervals of the variables associated with stone-free rate and residual stone.

Staghorn stones and larger stone size are commonly reported factors associated with lower SFR in both MPCNL [11,12,13,19] and PCNL [20,21,22]. In Zhu et al. (2011), larger stone size, multiple and staghorn calculi, location in the calix, and moderate to severe hydronephrosis decreased the chance for SFR [12]. Hydronephrosis was not found to be a significant factor in our study. However, the difference may be due to the fact that, in Zhu et al., hydronephrosis was an ordinal variable indicating levels of severity; whereas in our study, it was binary. In our data, 40 out of 45 stones in the calices were located in the lower calix, which may explain why this location was not associated with an increased risk for residuals. The forest plot of the odds ratios for the variables associated with a higher chance for SFR, an increased risk for residual stone, and those with no influence are given in Figure 4.

Operative duration was approximately 20 min longer for the patients with residual stone in our study. In the univariate analysis, BMI, stone location, size, staghorn, density, and volume were significantly associated with a longer surgery time. Among them, staghorn stones showed the longest operative duration (median 92 min), as compared to all other variables. Together with stone volume, they accounted for 39.00% of the variance in operative duration. In the meta-analysis conducted by Wu et al. (2021), the authors observed that staghorn and multiple stones were associated with longer operative time in MPCNL, as compared to PNCL. Such stones took longer time to break into smaller pieces in MPCNL, produced more debris, and prolonged the time to retrieve the stones [8].

In comparative research, shorter hospital time is reported as an advantage of MPCNL over PCNL [8]. This is attributed to a higher postoperative tubeless rate and/or a smaller nephrostomy tube diameter, which are associated with a shorter recovery time [22,23,24,25,26]. In our data, the median hospital stay was approximately 4 days and did not show large individual variations. The factors that were significantly associated with longer hospitalization time were the presence of hydronephrosis and staghorn stones.

Post-operative complications on the Clavien-Dindo scale were rare and predominantly of Grade 1. We did not find a link between their occurrence and the outcome of MPCNL or any of the patient/stone characteristics that were reported in other studies, such as ASA scores, staghorn stones, multiple tracts, and longer surgery duration [6]. The only factor with an impact was the presence of hydronephrosis, which was associated with a higher incidence of complications, including the few complications of Grade 2 or 3. This fact explains the longer hospitalization time that was characteristic of the patients with hydronephrosis.

## 5. Conclusions

By including data from a population of Bulgarian patients, the current study adds to the external validity of the conclusions made by prior studies on MPCNL in the treatment of renal stones. We have contributed to the scientific effort to identify risk factors that may decrease the chance for immediate stone-free outcome, necessitate longer operative time, increase the risk for complications, and lead to longer hospitalization. We have provided algorithms that can be used in clinical practice for making informed decisions and setting realistic expectations about each patient. In the studied population, staghorn stones and stones in more than one location were the strongest risk factors for residual stone. Staghorn stones and larger volume were linked with a longer operative duration. The presence of hydronephrosis increased the risk of complications and longer hospitalization.

## Figures and Tables

**Table 1:** Demographic and clinical characteristics.

Variables	All Patients	Groups		
		Stone-Free *n* = 136	Residual Stone *n* = 27	*p*
Age: Mean ± SD	53.60 ± 13.47	54.14 ± 13.59	50.85 ± 12.79	0.246 ^t^
Sex: *n* (%)				
○ Male	103 (63.00%)	85 (62.50%)	18(66.70%)	0.828 ^f^
○ Female	60 (37.00%)	51 (37.50%)	9 (33.30%)	
BMI: *n* (%)				
○ Normal	30 (18.40%)	26 (19.10%)	4 (14.80%)	0.778 ^χ2^
○ Overweight	81 (49.70%)	66 (48.50%)	15 (55.60%)	
○ Obese	52 (31.90%)	44 (32.40%)	8 (29.60%)	
Operative risk: *n* (%)				
○ ASA 1	54 (33.00%)	45 (33.10%)	9 (33.30%)	0.972 ^χ2^
○ ASA 2	39 (24.00%)	33 (24.30%)	6 (22.20%)	
○ ASA 3	70 (43.00%)	58 (42.60%)	12 (44.50%)	
Hematuria history: *n* (%)	36 (22.00%)	31 (22.80%)	5 (13.90%)	0.801 ^f^
Hydronephrosis: *n* (%)	96 (58.90%)	79 (58.10%)	17 (63.00%)	0.675 ^f^
HGB g/L (Mean ± SD)	138.27 ± 14.19	138.27 ± 14.20	138.25 ± 14.42	0.995 ^t^
WBCs g/L (Mean ± SD)	8.76 ± 2.67	8.81 ± 2.72	8.50 ± 2.43	0.583 ^t^
PLT mc/L (Mean ± SD)	269.30 ± 80.46	268.39 ± 74.45	273.88 ± 107.39	0.747 ^t^
INR (Mean ± SD)	0.99 ± 0.05	0.99 ± 0.05	0.99 ± 0.02	0.914 ^t^
Creatinine mmol/L (Mean ± SD)	92.19 ± 22.98	92.72 ± 23.30	89.55 ± 21.53	0.515 ^t^
Urea mmol/L (Mean ± SD)	5.64 ± 1.36	5.63 ± 1.33	5.67 ± 1.53	0.875 ^t^
Glucose mmol/L (Mean ± SD)	5.94 ± 1.44	5.96 ± 1.46	5.86 ± 1.36	0.733 ^t^
Hospital stay (days) (Median (IRQ)	4 (2)	4 (2)	5 (3)	0.123 ^U^
Operative duration (minutes) (Mean ± SD)	61.91 ± 26.29	58.55 ± 23.93	78.81 ± 31.26	<0.001 ^t^

HGB—Hemoglobin; WBCs—Leucocytes; PLT—Thrombocytes; INR-International normalized ratio; ^t^—independent samples *t*-test; ^f^—Fisher’s exact test; ^χ2^—chi-square test; ^U^—Mann–Whitney U test; IRQ—interquartile range.

**Table 2:** Stone characteristics across the stone-free and residual-stone groups.

Parameters	Total	Groups		
		Stone-Free	Residual Stone	*p*
Burden: *n* (%)				
○ Solitary	113 (69.30%)	113 (83.10%)	0 (0.00%)	<0.001 ^χ2^
○ Multiple	34 (20.90%)	22 (16.20%)	12 (44.40%)	
○ Staghorn	16 (9.80%)	1 (0.70%)	15 (55.60%)	
Location: *n* (%)				
○ Pelvis	72 (44.20%)	71 (52.20%)	1 (3.70%)	<0.001 ^χ2^
○ Calices	45 (27.60%)	42 (30.90%)	3 (11.10%)	
○ Both	46 (28.20%)	23 (16.90%)	23 (85.20%)	
Measurements: Mean ± SD				
○ Size (mm)	12.29 ± 6.15	11.06 ± 4.78	21.94 ± 10.19	<0.001 ^t^
○ Volume (cm^3^)	1.57 ± 3.18	0.98 ± 1.42	4.55 ± 6.43	0.008 ^t^
○ Density (HU)	811.49 ± 278.15	779.86 ± 284.49	1070.92 ± 276.15	<0.001 ^t^

^χ2^—chi-square test; ^t^—t-test.

**Table 3:** Results from the multivariate binary logistic regression for factors that are significantly associated with a residual-stone outcome.

Predictors	B Coefficient	SE	Wald Statistics	*p*	Odds Ratio (95% CI)
Constant	–8.17	1.74	21.89	<0.001	
Stone size	0.10	0.04	4.11	0.04	1.12 (1.006 to 1.25)
Stone density (HU)	0.002	0.001	4.78	0.02	1.03 (1.005 to 1.06)
Stone burden					72.48
Staghorn (1)	4.28	1.29	10.99	<0.001	(5.76 to 91.81)
Stone location					23.91
Pelvis & calices (1)	3.17	0.89	12.64	<0.001	(4.15 to 107.56)

Dependent variable: immediate postoperative MPCNL outcome (1 = residual stone; 0 = stone-free); Stone burden included two categories: staghorn = 1; single or multiple stones = 0. Stone location was coded: 1 = pelvis and calices; 0 = either pelvis or calices.

**Table 4:** Factors influencing operative duration and hospital stay.

Factors	Operative Duration (minutes)	*p*	Hospital Stay (days)	*p*
Median (IQR)				
BMI				
○ Normal	47.00 (19.00)	0.037	4.50 (4.00)	0.714
○ Overweight	56.00 (41.50)		5.00 (3.00)	
○ Obese	54.00 (35.75)		4.00 1.75)	
Operative risk				
○ ASA 1	50.00 (23.25)	0.251	4.00 (3.00)	0.039
○ ASA 2	53.00 (32.00)		4.00 (2.00)	
○ ASA 3	55.50 (51.25)		5.00 (3.00)	
Stone location				
○ Pelvis	49.00 (35.00)	0.018	4.00 (3.00)	0.984
○ Calices	53.00 (26.50)		4.00 (2.00)	
○ Both	60.00 (55.50)		4.00 (2.35)	
Stone size				
○ Small (<10 mm)	45.00 (37.75)	<0.001	4.00 (3.00)	0.624
○ Medium (10–20 mm)	55.00 (28.00)		4.00 (2.50)	
○ Large (>20 mm)	83.50 (44.25)		5.00 (2.00)	
Stone burden				
○ Solitary	50.00 (31.50)	0.001	4.00 (2.00)	0.008
○ Multiple	55.00 (57.75)		4.00 (3.00)	
○ Staghorn	92.00 (47.75)		6.00 (2.75)	
Hydronephrosis				
○ Yes	55.00 (46.00)	0.304	5.00 (3.00)	0.020
○ No	52.00 (26.00)		4.00 (1.00)	
Correlation coefficients (rs)				
Stone density (HU)	0.252 (95% CI: 0.099 to 0.3920)	0.001	0.020 (95% CI: −0.134 to 0.173)	0.798
Stone volume	0.471 (95% CI: 0.333 to 0.587)	<0.001	0.099 (95% CI: −0.056 to 0.249)	0.209

Kruskal–Wallis test with post-hoc pairwise comparisons was used for more than two categories; MW—Mann–Whitney U test was used for comparison of two categories; rs—Spearman rank-order correlation.

## Data Availability

The data presented in this study are available on request from the corresponding author. The data are not publicly available due to institutional restrictions.
